# Towards comprehensive clinical trials for new tuberculosis drug regimens: policy recommendations from a stakeholder analysis

**DOI:** 10.1136/bmjgh-2023-014630

**Published:** 2024-04-22

**Authors:** Simone Villa, Pierpaolo de Colombani, Lucia Dall’Olio, Giuliano Gargioni, Mario Raviglione

**Affiliations:** Centre for Multidisciplinary Research in Health Science, University of Milan, Milano, Italy

**Keywords:** Tuberculosis, Clinical trial, Health policy

## Abstract

**Background:**

Research and development (R&D) of new drugs and regimens against tuberculosis (TB) is evolving to meet new challenges and face limited investments in the sector. To effectively improve and fill existing gaps, researchers and trialists should engage a broad spectrum of stakeholders. With this study, we aim to map the interests in TB R&D raised by the main stakeholders in the TB field.

**Methods:**

We conducted semistructured, short interviews to gather insight and viewpoints on innovation on TB drugs and regimens R&D of policy-makers, national TB programme officers, donors, funders, non-governmental organisations and research institutions.

A composite measure of the relevance of topics that emerged was computed by implementing different models considering the importance for researchers and the urgency to implement those changes during the trial, the number of citations each topic received, and the maximum value of the influence of stakeholders who had raised the topic.

**Results:**

50 stakeholders, out of 56 identified, were interviewed and almost half were policy-makers and governmental institutions. Several stakeholders highlighted the importance of disseminating information about clinical trials’ methodology and emerging preliminary results, followed by the need to pursue early discussion around access and pricing of safe and effective TB innovations, although different categories of stakeholders prioritised different topics. Using different methods for ranking topics, the results remained almost unchanged. Notably, post-trial operational research ranked higher in models with higher weight for the parameter considering the number of citations.

**Conclusion:**

Researchers and research consortia embarking on phase 2 and 3 clinical trials should consider a broad set of elements when planning and designing trials’ protocols, all aiming at lowering the price and improving access to emerging TB innovations, besides meeting regulatory criteria. This can only be achieved by consulting and engaging relevant stakeholders in the discussion.

**WHAT IS ALREADY KNOWN ON THIS TOPIC**

- Existing gaps between the development of tuberculosis (TB) innovations and their adoption—generally after more than a decade—in high TB countries are well known. This is a general phenomenon concerning all innovations in diseases disproportionally affecting low-income and middle-income countries (LMICs).

**WHAT THIS STUDY ADDS**

- By consulting 50 international and national stakeholders, we derived a set of 10 recommendations aimed at improving clinical trials for TB drugs.

**HOW THIS STUDY MIGHT AFFECT RESEARCH, PRACTICE OR POLICY**

- Our study provides elements and actions that researchers and consortia could implement at the beginning of clinical trials to provide equitable and rapid access to TB innovations being developed. The proposed recommendations apply to development of innovations for all diseases disproportionally affecting LMICs.

## Introduction

During the last decade, drug research and development (R&D) for tuberculosis (TB) has moved its focus from developing single antimicrobial agents to combination treatment regimens containing both new compounds and key existing or repurposed drugs. This approach results in the introduction of completely new regimens rather than the addition of a single drug to previously existing regimens, thus helping to prevent the rapid onset of drug resistance.^1^ This approach could further allow better management of sample size of clinical trials and important reductions in study time and costs.^2 3^

However, currently, when new TB drugs or regimens are developed and licensed for marketing, additional information is required to facilitate endorsement by policy-making bodies and to support rapid and effective implementation in countries.^4 5^ For example, bedaquiline was licensed after a phase 2 clinical trial in 2012 but recommended as an ‘add-on agent’ only in 2016 and included in WHO-recommended shorter regimens only in 2019.^1 6 7^ Such experience, complementing the lessons learnt from the R&D of COVID-19 vaccines, suggests that new approaches in R&D are urgently necessary and feasible in TB.^8 9^

Therefore, in pursuing R&D for new TB regimens, researchers working on clinical trials must effectively and timely engage a broad range of stakeholders who consider the cascade of events ultimately resulting in enhanced access to new treatments for all. This includes those in charge of designing and conducting research, but also those who will play a key role in the implementation of the innovations emerging from the trials. Past delays in transferring new tools from high-income countries capable of affording them to less economically developed countries have several explanations: problems with local registration,^1 10 11^ need for testing locally,^12–14^ lack of endorsement by entities such as WHO,^1 5 15 16^ high price,^17–19^ constraints in procurement and supply management,^5 20 21^ limited manufacturing capacity,^4 22 23^ as well as lack of awareness and false perceptions on potential adverse reactions and other complications.^24 25^ Therefore, engaging key stakeholders from the first step of designing a clinical trial may facilitate and accelerate the future uptake of new TB regimens in any country, especially the low-income and middle-income countries (LMICs) with high TB burden. While these principles emerge clearly from R&D efforts in TB, rapid implementation of innovations is a general concern to be addressed for any high-burden disease.^5 26 27^

An assessment of the cascade between a new drug or regimen developed and its full access by people with TB allows to identify in advance all risks of possible attrition and delay and possible solutions to mitigate them. In the case of TB drugs and regimen, most countries today prefer to make decisions on national standards and policies in line with WHO recommendations.^17^ These decisions require a careful scrutiny of clinical and preclinical evidence—as done by the regulatory agencies—as well as the consideration of a broader set of criteria that are part of the GRADE (Grading of Recommendations, Assessment, Development and Evaluations) evidence generation system adopted by WHO.^1 28–30^ Such framework builds on information about efficacy and safety of the drugs and regimens that are complemented by considering service providers’ and beneficiaries’ views, feasibility and acceptability, and economic assessments.

Therefore, it is imperative for any clinical trial effort to identify the challenges in the uptake of TB treatment innovations as early as possible. This means, in other words, having a profound knowledge of the steps of such uptake in all countries.

This article reports on an initial stakeholder analysis conducted to assess their opinions about the clinical trial approach proposed by UNITE4TB which, by applying a strict methodology, has produced policy recommendations for future pursuance and lessons learnt for new clinical trials to be conceived and undertaken. We believe that the principles and conclusions from this paper are of value beyond the TB field.

## Methods

This analysis has been conducted as part of the activities under the project UNITE4TB (https://www.unite4tb.org/) to engage main TB stakeholders in the world to increase their awareness and collect their comments during the implementation of clinical trials with a mind on when the new TB regimens will be available and ready for country uptake.^31^

From March to August 2022, we organised 1-hour, online, semistructured interviews with our key informants, to brief on the UNITE4TB project and discuss foreseen challenges in the implementation of the clinical trials and challenges in the future uptake of new TB treatments. We selected the key informants from a list of well-known TB stakeholders of national and international leverage among policy-makers and governmental institutions, including national TB programmes (NTP), donors and funders, non-governmental organisations (NGOs) and research entities.

### Criteria for inclusion of stakeholders

Stakeholders are those actively engaged in national or international response to TB. They represent an ideal variety of views (due to their mandate, funding, constituencies, area of operations and experience) in R&D, financing, health policy-making and service delivery.

### Criteria for exclusion of stakeholders

To minimise potential information biases, we excluded the stakeholders who are members of the UNITE4TB consortium. We also excluded the European Medicines Agency (EMA), academic institutions and civil society organisations.

### Interview invitation

Our team working within the Centre for Multidisciplinary Research in Health Science (MACH) of the University of Milan (Milan, Italy) initially contacted the stakeholders selected via email, providing a short description of the UNITE4TB objectives and proposing a 1-hour online interview meeting in a following day of choice. The participation of other relevant local partners at stakeholder’s choice was welcome.

### Interview process

We started our meetings with a short introduction of the participants and a description of the scope of the interview. We then proceeded with a standard presentation, given by one member of our team, on the UNITE4TB project, its objectives and initial approaches in conducting phase 2 clinical trials.

### Data collection

The main points from each interview were summarised in a note for the record drafted by the MACH team and subsequently shared with the stakeholders for input before finalisation. The information contained in the note for the record was further analysed and summarised by two independent persons (LD and SV) in a predefined Microsoft Excel file. Any disagreement in the analysis of the notes for the record was resolved by discussion among the two authors.

### Variables

Stakeholders were categorised as follows: (1) policy-makers and governmental organisations, (2) donors and funders, (3) NGOs and (4) research institutions (as per online supplemental tables S1–S4). Furthermore, we classified each of them by their level of operations and/or mandate (ie, international or national). Both categories of stakeholder and level of operations were used to group stakeholders according to different levels of influence (active or passive) on decision-making and implementation processes related to the introduction of new drugs and treatment regimens (online supplemental table S5).

10.1136/bmjgh-2023-014630.supp1 Supplementary data

Each topic emerging from the interviews was classified by its relevance (online supplemental table S6) and urgency in relation to a phase 2B/C clinical trial (online supplemental table S7). Furthermore, to facilitate assessment, topics were grouped into three categories, that is, clinical trial design and sites, access and uptake, and collaboration, by two independent authors (LDO and SV).

### Topic interest

The level of urgency (*u*) and importance (*j*), together with the maximum value of the influence of stakeholders who had raised the topic (*γ*) and the number of times that the topic was mentioned by stakeholders (*m*), were used to compute a composite measure of priority ( P ). Different models were used and compared as described in online supplemental table S8. The list of topics emerging from the discussions and corresponding computed priority scores were stratified by the type of stakeholders involved to better define the importance of the topic according to the different stakeholder category.

Data were analysed by using R V.4.2.2 (packages tidyverse, dplyr, devtools, ggplot2, ggpubr, highcharter and maps).

Results were presented and discussed internally with the UNITE4TB Project Executive Team, which comprises pharmaceutical company representatives, to gather expert opinions on resulting recommendations and conclusions.

## Results

Among the 56 stakeholders contacted for an interview, 4 declined and 2 did not reply, resulting in a final list of 50 stakeholders interviewed (89.3%). Those who declined the interview were one governmental programme, two research institutions and one international donor. The representative of one international policy-maker and one NTP never replied.

Among the 50 stakeholders interviewed, 27 (48.2%) were representatives of policy-making and governmental institutions (eg, WHO offices and NTPs), 9 (16.1%) of donors and funders, 10 (17.8%) of NGOs and 4 (7.1%) of research entities. The representatives of the 19 NTPs interviewed (38% of all stakeholders) were from countries located in all 6 WHO regions. These countries were responsible for 8 (75%) of the 10.6 million estimated TB incident cases globally (online supplemental figure S1). 13 and 11 NTPs, respectively, were from countries suffering from the highest burdens of multidrug-resistant/rifampicin-resistant TB and HIV-associated TB.

### Key topics raised by stakeholders

25 different topics were identified during the interviews with stakeholders as shown in table 1.

**Table 1:** Overall topics emerged and corresponding level of emergency and importance, and the priority score computed using different models

#*	Topic	Type	Urgency	Importance	No. citations	Priority score		
						Mod-PA	Mod-PB	Mod-PC
1	Disseminate CT information	Collaboration	Medium	High	26	0.98	1.00	1.00
2	Access and pricing	Access and uptake	Medium	High	22	0.94	0.94	0.94
3	CT sites	CT elements	High	Medium	21	0.93	0.92	0.92
4	Country uptake	Access and uptake	Medium	High	20	0.91	0.91	0.90
5	Coordination among consortia	Collaboration	High	High	12	0.94	0.88	0.83
6	Operational research	Access and uptake	Low	High	14	0.73	0.72	0.70
7	Target product profile	CT elements	High	High	5	0.86	0.78	0.70
8	WHO requirements	Access and uptake	High	High	5	0.86	0.78	0.70
9	Regulatory requirements	Access and uptake	High	Medium	8	0.78	0.72	0.67
10	Special population	CT elements	High	High	3	0.83	0.75	0.66
	CT design	CT elements	High	Medium	6	0.75	0.69	0.63
	Adherence	CT elements	High	Low	8	0.66	0.62	0.59
	Data quality	CT elements	High	Medium	3	0.72	0.65	0.57
	Economic analysis	CT elements	Medium	High	3	0.72	0.65	0.57
	Microbiology biobank	CT elements	High	Medium	1	0.69	0.62	0.54
	Biomarkers	CT elements	High	Low	5	0.62	0.58	0.53
	FDC	Access and uptake	Low	High	4	0.61	0.56	0.51
	Coordination with professional organisations	Collaboration	Medium	Medium	1	0.58	0.52	0.45
	Essential drug list	Access and uptake	Low	High	2	0.56	0.50	0.44
	AI/ML	CT elements	High	Low	3	0.53	0.48	0.42
	Coordination between community organisations	Collaboration	Medium	Medium	1	0.51	0.45	0.39
	Capacity building	Access and uptake	Medium	Low	3	0.45	0.41	0.37
	Phase 3	Access and uptake	Low	Low	4	0.38	0.36	0.34
	Phase-out and phase-in	Access and uptake	Low	Medium	2	0.41	0.36	0.32
	Emergence of AMR	Access and uptake	Low	Low	4	0.35	0.33	0.31

*Topics are ranked based on model Mod-PC.

AI/ML, artificial intelligence/machine learning; AMR, antimicrobial resistance; CT, clinical trial; FDC, fixed-dose combinations; Mod-PA, Model priority A; Mod-PB, Model priority B; Mod-PC, Model priority C.

#### Clinical trial design

Regarding selection of study sites, 21 stakeholders interviewed highlighted the importance for clinical trials not just to consider the inclusion of TB high-burden countries but also to engage those with specific requirements for drug licensing and approval (eg, China and India) and those with a variety of implementation challenges (eg, hard-to-reach areas, ethnical and culture differences). They suggested consideration for the availability of local research and laboratory capacities and the presence of ongoing clinical trials with potential competitive overlapping.

Eight stakeholders underlined the relevance that clinical trials in TB use tools for enhancing TB treatment adherence to limit the emergence of drug resistance to newly introduced drugs.

Different stakeholders recommended that clinical trials should be designed not only to meet the regulatory requirements for new drug licensing and marketing authorisation but also to include the endpoints needed by policy-makers to support their recommendations. For example, WHO’s Global TB Programme suggested being consulted early in the design of large clinical trials to facilitate, in later stages, a rapid transition from approval of newly developed TB drugs and regimens to policy recommendations and guidelines.

Furthermore, five stakeholders mentioned that clinical trials should adhere to TB target product profiles (TPPs) for TB treatment regimens, besides satisfying regulatory requirements for drug approval. This should also be considered early in the conduct of a trial because TPPs are sensitive to costs, accessibility and uptake by countries. To facilitate such a transition, three stakeholders mentioned that phase 2 clinical trials should incorporate some economic elements to allow preliminary cost-effectiveness and cost–utility analyses. This is often a concern in late phase 3–4 clinical trials, but the acceleration of formulation of new policies and guidelines as well as their implementation and country uptake require an early economic assessment to influence the sustainability of the policies adopted and decisions by international donors to support innovations.

#### Equitable access and uptake of new TB innovations

Among topics classified as relevant to access and uptake of innovations from TB clinical trials, 22 stakeholders expressed the importance of phase 2 clinical trials to pursue early discussions on access with other research consortia, pharma companies, agencies and donors. In LMICs and any high TB burden countries, affordability and pricing are key challenges when pursuing adoption of new TB drugs and regimens as well as related diagnostics (eg, drug-susceptibility testing for drug resistance monitoring). 20 stakeholders expressed concern that such countries will have to face major hurdles to ensure rapid uptake if proper actions, such as the ones proposed in table 2, are not undertaken.

**Table 2:** Challenges and proposed actions identified by stakeholders for the rapid uptake of new TB drugs and regimens by countries

Challenge	Proposed actions
Costs	Initiate discussions between companies, research consortia and policy-makers
WHO reccomendation	Incorporate WHO requirements in CT design
Agreement with donors	Initiate discussions with potential donors and funders
Coordination with NTPs	Engage NTPs early in the development of CT
Country-specific registration requirements	Engage NTPs early in the development of CT
Manufacturing capacity	(Engagement with NTPs might help)
Fixed dose combinations	Initiate discussions between companies, regulatory agencies and policy-makers
Laboratory capacity	Engage NTPs early in the development of CT and foster in-country capacity building
Ad hoc monitoring tools	Develop guidance to share once new TB drugs and regimens are avaialble

CT, clinical trial; NTP, national TB programme; TB, tuberculosis; WHO, World Health Organization.

A key step to move from drug R&D to their quick adoption in countries is their approval by regulatory agencies. Eight stakeholders mentioned that some national regulators require clinical trials to be conducted in their own country and include their populations and settings. The absence of local evidence may significantly delay the uptake of innovation in countries such as China and India, with a large number of people affected by TB and their major impact on the global market.

Furthermore, even after approval by the most stringent regulatory authorities (eg, Food and Drug Administration (FDA) and EMA) stakeholders suggested that most countries await WHO’s recommendations on the use of new TB drugs and regimens to make decisions on adoption and prepare national guidelines. Some countries require extra steps such as the inclusion of new TB drugs in the WHO’s essential medicine list or the conduct of cost-effectiveness/benefit analyses. The latter is necessary to demonstrate that costs of new treatments are balanced by the level of treatment success improvement and the number of beneficiaries, as exemplified by South Africa. 14 stakeholders proposed the idea of early planning of operational research before the approval of stringent regulators to identify and address implementation challenges.

#### Collaboration among large clinical trials

More than half of the stakeholders interviewed (n=26) reported the importance of the early engagement of key stakeholders and other trial consortia to share methodology and goals so that awareness can amplify and further disseminate information. 12 stakeholders further underlined the need for technical coordination and synergy among different research consortia, especially the largest ones, to avoid duplication of efforts, competition for clinical trial sites and waste of limited financial resources. Coordination should not be limited to the consortia leading clinical trials on TB treatments but expanded to other initiatives such as specimen biobanks, biomarkers as surrogate endpoints in clinical trials, economic analysis and operational research.

Finally, the importance that researchers, research institutions and pharma companies involved in clinical trials should engage and coordinate with professional societies (n=1) as well as with community representatives and organisations (n=1) was emphasised.

### Topic priority scoring

Using the priority score as computed in online supplemental table S8, all models (model priority [Mod-P] A, B, and C) followed the frequency of citations of a topic by stakeholders with few exceptions. All models prioritised the dissemination of sharing clinical trial information (eg, methodology, trial design, compounds and sites) with external stakeholders.

By focusing on the top 10 topics based on model Mod-PC, little difference was observed across different models adopted.

Stratifying by the type of stakeholders, policy-makers and governmental institutions (online supplemental table S9) share the same top 10 priorities as the general figures (table 1). Donors and funders (online supplemental table S10) valued particularly compliance of clinical trials to TPPs for TB regimens, data quality and economic analysis. Likewise, adherence to TPP was further considered important by NGOs that were also concerned about specific requirements by national regulatory authorities (online supplemental table S11). Research institutions valued the importance of including research on biomarkers as surrogate endpoints in clinical trials during the development phase. They also emphasised the need for clinical trials to complete phase three before approval by stringent regulatory authorities (online supplemental table S12).

## Discussion

Our stakeholder analysis was a major instrument to inform key TB stakeholders about the UNITE4TB and to gather their viewpoints and priorities when dealing with TB innovations emerging from clinical trials.

Among the 25 topics raised, the top 4 were the need to ensure dissemination of methodology and results (raised by 26 stakeholders, 52%), access and pricing (22, 44%), selection of clinical trial sites (21, 42%) and country uptake (20, 40%). Policy-makers and governmental institution mostly highlighted the need for early dissemination of methodology of clinical trial as well results as soon as they emerge (13, 48.2%), and the importance of selection of clinical trial sites (12, 44.4%). Donors were mainly interested in access to, and pricing of, new innovations resulting from clinical trials (8, 88.9%) and in rapid country uptake of such innovations (6, 66.7%). The latter was also the main topic (6, 60.0%) raised by NGOs together with the dissemination of clinical trial methodology and results (6, 60.0%). Research institutions focused mainly on the decision on clinical trial sites (4, 100.0%) and dissemination of clinical trial methodology and results (3, 75.0%).

Using different methods for ranking, the top 10 topics remained almost unchanged except for the 2 models (ie, PB) in which the need to conduct post-trial operational research ranked lower. In those models, actually, the parameter considering the number of stakeholders citing each topic weighted less (ie, $\frac{0.3m}{m_{max}}$ for PA) than the other two models (ie, $\frac{0.4m}{m_{max}}$ for model PB and $\frac{0.5m}{m_{max}}$ for PC).

All information gathered during discussions with stakeholders in this project can be used, ultimately, to draw key recommendations for research consortia and trialists involved in TB drugs R&D. Successful conceptualisation and conduct of a clinical trial aiming at rapid implementation of, and access to, emerging innovations require some revisiting of traditional approaches limited to demonstration of safety and efficacy of any new agent.

Currently, after development, new TB drugs and regimens are not automatically adopted by countries even after authorisation from stringent and worldwide recognised regulatory agencies^5 17^ as most countries, especially LMICs, today make decisions on national standards and policies awaiting, and aligning to, WHO recommendations.^15^ These decisions require a careful scrutiny of clinical and preclinical evidence—as done by regulatory agencies—as well consideration of a broader set of criteria.^32^ This means that clinical trials and research consortia, as clearly shown in our analysis, will benefit from early and close collaboration with WHO if TB innovations resulting from their efforts are to be rapidly taken up by countries. The evaluation of new regimens by WHO uses mainly the GRADE system.^28^ The GRADE system considers the efficacy and safety of new drugs and regimens, as also assessed by stringent regulatory agencies (eg, EMA and FDA), as well as TB service providers’ and beneficiaries’ views, feasibility and acceptability aspects and economic assessments.^1 28–30^

The price of new TB tools, such as TB drugs and regimens as well as related diagnostics to monitor the emergence of drug resistance, resulting from clinical trials is one of the challenging concerns raised by stakeholders when deciding future uptake by LMICs.^4 18 24 33^ It is a highly sensitive issue that requires early discussion between pharmaceutical companies and key international stakeholders. This is a necessary step to ultimately ensure equitable access to life-saving tools all in all settings as promoted within the principles of universal health coverage. Dependence on international funders (eg, the Global Fund to Fight AIDS, TB and Malaria) by many LMICs is a challenge to be tackled. Cost–utility analyses of new TB treatment regimens are a tool to guide long-term sustainable choices by governments and their external funders.^34 35^

Because of low investments in TB R&D,^36^ it is essential to minimise efforts required to deliver safe and effective TB innovations through, for example, the establishment of a global network of clinical trial sites ready to run phase 2 clinical trials based on effective information sharing among trialists. Both solutions require the development of formal collaborations and partnerships among main research consortia involved in TB drug R&D thus including pharmaceutical companies. The presence of many research consortia active in TB R&D with similar aims and composition was the object of curiosity and concern of many of the stakeholders interviewed.^37^ It was evident to everybody that the common good should prevail over a single consortium’s agenda and that collaboration/coordination among consortia should start immediately to adjust plans towards global synergy in research. At present, there is a perceived risk of duplication of efforts and potential overlapping of clinical trial sites.

To be effective, potential clinical trial sites for phase 2 should include several of the largest TB high-burden countries and a wide range of different epidemiological scenarios and field implementation challenges, as well as the presence of different populations at risk of TB. The availability of capacities and resources to meet sound research criteria should also be considered. The feedback received from stakeholders also emphasised the key importance to include sites in countries (eg, China and India) where registration of new drugs requires the conduct of trials among their own populations. This will avoid major delays in introducing innovations in their markets with eventually an impact on uptake worldwide and, hopefully, on national and global TB burden.^12 38 39^

Due to the complex tasks mentioned, it is key that clinical trials and research consortia inform, engage and collaborate with NTPs, an important and usually neglected issue, especially in high TB burden countries and where clinical trial sites are located. NTPs have a major role in policy-making at national level and adoption of new tools in routine TB control activities. Their frequent lack of information on clinical trials goals and aims detected in our analysis is a major concern to be addressed systematically.^40 41^

Effective engagement of public health authorities, from WHO to NTPs, is of utmost importance to minimise rapid emergence and spread of drug resistance soon after the introduction of new agents. This concern was repeatedly raised by stakeholders. Some suggested that promising new TB drugs part of a regimen should be registered together with consideration for manufacturing of fixed-dose combinations (including for paediatric formulations) and for operational research on treatment adherence.

Our study reiterates WHO’s call to enhance research and innovations as part of the End TB Strategy, with special attention to international collaboration, guarantee of long-term funding, introduction of new and affordable technologies, and inclusion of subpopulations with different vulnerabilities.^42 43^ Towards these aims, the Unite4TB Project, that is, a consortium of public and private partners, has included a work package specifically to engage TB stakeholders, including NTPs, from the ve4rz first year of the project. Nevertheless, after almost a decade from WHO’s promotion of those principles, the establishment of national partnerships between NTPs and other research institutions and groups has not yet been optimised. Such collaborations and networking have the potential to enable NTPs to actively contribute to research consortia on matters like trial design, clinical trial site selection, capacity building and technology transfer. As recommended previously,^42 43^ is, therefore, crucial that NTPs and Ministries of Health develop clear, structured and financed national TB research plans to pursue strategic discussions with international funders and research consortia that are usually from ‘Global North’ institutions.

Our study has several limitations. First, on several occasions more than one person with different affiliations was interviewed per each institution at the same time. This, on one hand, may improve the diversification and contents of the discussion; on the other hand, the power and influence of opinion of each agency interviewed could be diluted. For instance, in some interviews, the NTP manager and a consultant or donor could be present at the same time. To avoid unbalances across stakeholders, we have decided not to account for the power sometimes used in stakeholder analyses when computing topics’ priority score.^44^ Second, interviewers were conducting unstructured interviews with stakeholders to be able to capture different interests from interviewees. However, with time interviewers’ knowledge of the main issues raised by stakeholders increased and may have biased other interviewees in their comments. To correct this potential problem, interviewers tried to maintain a neutral position when asked for clarifications or comments. A third limitation was the modest inclusion of community organisations and of activist groups due to the existence of a specific community advisory group in UNITE4TB that provides regular feedback to trial leaders, However, we considered that some of the activist NGOs interviewed well represent the opinion of those communities affected by TB. A final limitation is the exclusion from the interviews of members of the consortium, including representatives of pharmaceutical companies or important international NGOs. However, these stakeholders were part of the internal governance of UNITE4TB and had the opportunity to provide their opinions during internal presentations of results. Those opinions were incorporated into the discussion of our findings.

In any case, our analysis demonstrates that most stakeholders clearly represent a fundamental resource for any ongoing and future clinical trial in TB and should be engaged in a wide range of coordination tasks depending on their scope and constituency. Among the stakeholders interviewed, there were those responsible for governmental funding, those essential in international policy-making and support, those members of different research consortia, those supporting NTPs in various manners, those with a consolidated credibility in the TB scientific community, and those working in civil society organisations. The vast majority of them were highly interested and available to collaborate and support clinical trials and research consortia involved in TB drug R&D, including UNITE4TB.

## Conclusions

To our knowledge, this is the first study of its kind exploring the perspectives and viewpoints around R&D of new TB drugs and regimens among the main stakeholders operating in the field of TB. To aggregate different issues raised by stakeholders, we have summarised them into ten priority recommendations (table 3) that should be adopted by any research consortium and researcher involved in R&D for new TB drugs and regimens. Beyond the field of TB, we also believe that any researcher embarking on phase 2 and 3 clinical trials work should consider, when planning and designing the trial and seeking funding, the importance of including new aspects of research that can be explored in parallel with the conduct of the trial. Those aspects, as we have shown in our study, are deemed crucial to ensure rapid implementation and future uptake of any innovations guaranteeing access to all. To effectively achieve this aim, the engagement of key stakeholders, the consultation for proper planning should become integral part of clinical trial protocols, especially when addressing research relevant to high-burden public health threats such as TB.

**Table 3:** 10 priority recommendations for future clinical trials in TB drug and regimen research and development

No.	Recommendation	Topic covered*
1	Ensure that clinical trial outcomes meet WHO requirements to develop policies and guidelines.	**Target product profile** **WHO requirements** **CT design** Economic analysis Data quality
2	Pursue strategic discussions to make costs of new treatment regimens, DST and other tests affordable to low-income and middle-income countries to guarantee equitable access to all in all settings.	**Access and pricing** **Country uptake** Essential drug list
3	Encourage development of fixed-dose combinations to be presented to regulatory authorities as soon as a new regimen is approved.	**Regulatory requirements** FDC Adherence Emergence of drug resistance
4	Strengthen coordination among TB clinical trial research consortia to limit duplications of R&D efforts.	**Coordination among consortia**
5	Engage major international stakeholders to facilitate dialogue among research consortia to coordinate efforts and solve bottlenecks in TB drug R&D and policy implementation.	**Coordination among consortia**
6	Carefully select clinical trial sites to include vulnerable and high-risk populations (eg, people living with HIV, children, MDR-TB).	**CT sites** **Special populations**
7	Enhance engagement of NTPs in the conduct of clinical trials to ensure proper selection of sites and to facilitate local research capacity.	**CT sites** **Country uptake** **Special populations** Operational research Capacity building
8	Support NTPs in identification of bottlenecks for rapid uptake of new regimen.	**Coordination between community organisations** **Country uptake** **Regulatory requirements** Adherence Coordination with professional organisations Capacity building Phase-out and phase-in
9	Advocate with investors and donors for adequate and stable funding of advanced clinical trial phases.	**Disseminate CT information** Phase three trial
10	Encourage effective, regular and timely information sharing with stakeholders of relevant scientific discoveries emerging from trials.	**Disseminate CT information**

*In bold are the topics with the highest priority score based on table 1.

CT, clinical trial; DST, drug susceptibility test; FDC, fixed-dose combinations; MDR, multidrug resistant; NTP, national TB programmes; R&D, Research and development; TB, tuberculosis.;

## Data Availability

Data are available on reasonable request. Deidentified data about the key words derived from interviews and models’ parameters are available upon request by emailing Dr SV (email: simone.villa@unimi.it).
