# Supplementary material for: Towards comprehensive clinical trials for new tuberculosis drug regimens: policy recommendations from a stakeholder analysis
Source: BMJ Glob Health. 2024 Apr 22;9(4):e014630. doi: 10.1136/bmjgh-2023-014630 (PMC11043750; doi:10.1136/bmjgh-2023-014630)
Supplement: Supplementary data [file bmjgh-2023-014630supp001.pdf]

Supplementary materials

Towards comprehensive clinical trials for new tuberculosis drug regimens: policy recommendations from a stakeholder analysis

Villa S, et al.

Table of Contents

Supplementary methods 2
Outcome variables 5
Supplementary results 6
Priority score 6

Supplementary methods

Stakeholders were categorized into four main categories:

1. Policymakers and governmental agencies;
2. Donors and funders;
3. Non-governmental agencies; and
4. Research institutions.

The full list of stakeholders is presented in **Table S1-S4**.

Table S1 List of Policymakers and governmental agencies identified.

Acronym	Stakeholder
**NTP Bangladesh**	National Tuberculosis Programme of Bangladesh
**NTP Brazil**	National TB Programme of Brazil
**NTLP Cambodia**	National Tuberculosis and Leprosy Programme
**NTP China**	National TB Programme of China
**NTP Ethiopia**	National TB Programme of Ethiopia
**NTP Georgia**	National TB Programme of Georgia
**NTP India**	National TB Programme of India
**NTP Indonesia**	National TB Programme of Indonesia
**NTP Kazakhstan**	National TB Programme of Kazakhstan
**NTP Moldova**	National TB Programme of Moldova
**NTLP Nigeria**	National Tuberculosis and Leprosy Programme of Nigeria
**NTP Pakistan**	National TB Programme of Pakistan
**NTP Philippines**	National TB Programme of the Philippines
**NTP Russia**	National TB Programme of the Russian Federation
**NTP South Africa**	National Tuberculosis Programme of South Africa
**NTP Tanzania**	National TB Programme of Tanzania
**NTLP Uganda**	National Tuberculosis and Leprosy Programme of Uganda
**NTP Uzbekistan**	National TB Programme of Uzbekistan
**NTP Viet Nam**	National TB Programme of Viet Nam
**US-CDC**	United States Centers for Disease Control and Prevention
**WHO-HQ**	Global TB Programme, World Health Organization
**WHO-AMRO**	TB Unit, Division of Communicable Disease Control, WHO, Regional Office for Americas
**WHO-EMRO**	TB Unit, Division of Communicable Disease Control, WHO, Regional Office for the Eastern Mediterranean
**WHO-EURO**	TB Unit, Division of Country Health Programmes, WHO, European Regional Office
**WHO-SEARO**	TB Unit, Division of Communicable Disease Control, WHO, Regional Office for South-East Asia
**WHO-WPRO**	End TB and Leprosy Unit, Division of Programmes for Disease Control, WHO, Regional Office for Western Pacific
**ECDC**	Disease Programme Unit, Division of Communicable Disease Control European Centre for Disease Prevention and Control

Table S2 List of donors and funders identified.

Acronym	Stakeholder
**FCDO**	Foreign, Commonwealth and Development Office
**IDPPR-GAC**	Infectious Diseases & Pandemic Preparedness and Response, Global Affairs Canada
**USAID**	United States Agency for International Development
**DFAT**	Department of Foreign Affairs and Trade
**GDF**	Global Drug Facility
**BMGF**	Global Health, Bill & Melinda Gates Foundation
**Global Fund**	The Global Fund
**Stop TB**	Stop TB Partnership Secretariat
**SIDA**	Swedish International Development Cooperation Agency
**Unitaid**	Unitaid

Table S3 List of non-governmental organizations identified.

Acronym	Stakeholder
**RIT-JATA**	The Research Institute of Tuberculosis, Japan Anti-Tuberculosis Association
**BRAC**	Bangladesh Rehabilitation Assistance Committee
**Damien**	Damien Foundation
**Dopasi**	Dopasi Foundation
**Union**	International Union Against Tuberculosis and Lung Disease
**MSF-France**	Médecins Sans Frontières of France
**PIH**	Partners in Health
**StopTB-IT**	StopTB Italia
**IFRC**	The International Federation of Red Cross and Red Crescent Societies
**LIGHT**	The Leaving no-one beHind, transforming Gendered pathways to Health for TB

Table S4 List of research organizations identified.

Acronym	Stakeholder
**EDCTP2**	European and Dev Countries Clinical Trials Partnership 2
**EDCTP3**	European and Dev Countries Clinical Trials Partnership 3
**NIH**	National Institutes of Health
**Rede TB**	Rede Brasileira de Pesquisa em Tuberculose
**Wellcome Trust**	Wellcome Trust

The countries whose national tuberculosis programmes (NTPs) were involved in this study are displayed in the map presented in **Figure S1**.

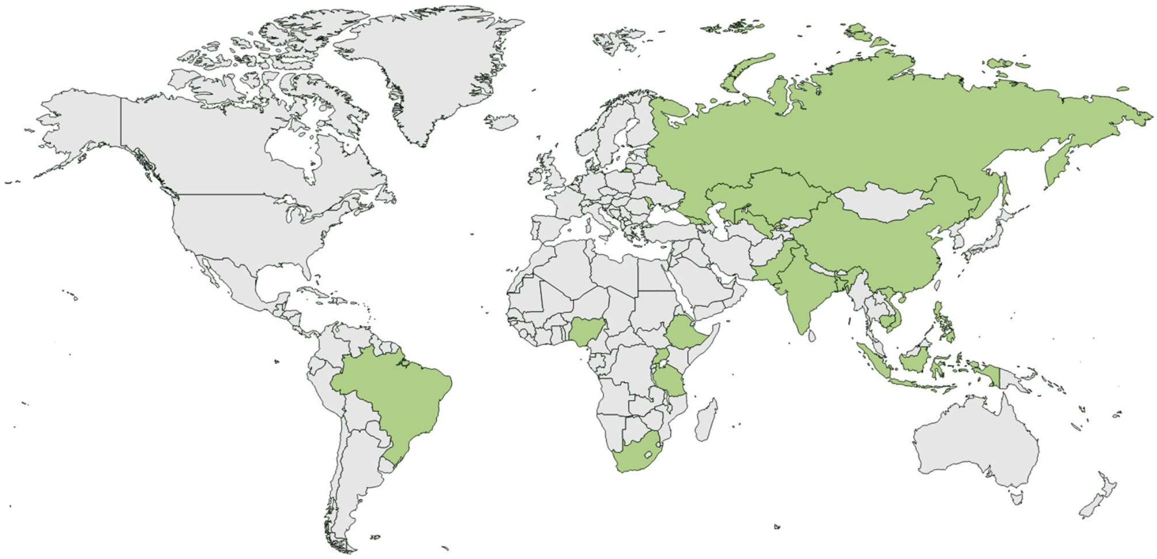

Figure S1 Geographical distribution of the national tuberculosis programmes interviewed.

All stakeholders were classified by the level of influence of their operations (**Table S5**).

Table S5 Influence levels.

Score	Level	Definition
**3**	**High**	Ability to affect consistently international and national policies, such as international policymakers and international donors (e.g., cooperation agencies), and national governmental implementers.
**2**	**Medium**	Ability to affect national policies, such as governmental funders (i.e., IDPPR-GAC, USAID, DFAT, FCDO, and SIDA), and others (i.e., GDF and StopTB Partnership).
**1**	**Low**	Contributing to the decision of national policies taken by others, such as NGOs and research institutes/networks.

Topics mentioned during interviews with stakeholders were classified by level of importance for researchers and trialists developing a Phase 2 clinical trial for TB drugs (**Table S6**) as well as for the deemed level of urgency (or timing) to implement those changes throughout a clinical trial (**Table S7**), by two independent authors (LD and SV).

Table S6. Topic importance.

Score	Level	Definition
**3**	**High**	A topic is defined as "high" when its application ensures that the aim of the project is achieved (necessary and sufficient).
**2**	**Medium**	A topic is defined as a "medium" when its application contributes to the achievement of the aim of the project, but it's not enough to ensure the project is achieved (necessary but not sufficient).
**1**	**Low**	A topic is defined as "low" when its application does not contribute to the aim of the purpose of the project (not necessary and not sufficient).

Table S7. Topic urgency.

Score	Level	Definition
**3**	**High**	A topic that has to be carried out in the "pre" phase of the project (during years 1 and 2 of the project) is classified as "high" urgency.
**2**	**Medium**	A topic that has to be carried out in the "during" phase of the project (during years 3 to 6 of the project) is classified as "medium" urgency.
**1**	**Low**	A topic that has to be carried out in the "post" phase of the project (after year 6 of the project) is classified as "low" urgency.

Outcome variables

For each topic raised by stakeholders, we computed a measure of the topic's importance for researchers and institutions working on Phase 2 clinical trials for TB (**Table S8**) by considering the number of citations (m), the level of importance (j) and urgency (u) of a topic based, only in the general model, by the maximum level of the stakeholders who have mentioned the topic (γ). Different models were created to weigh attributes differently and compare models' output.

The following formulas are weighted with values arbitrarily selected by the authors to account for a similar effect of the level of importance and urgency of a topic and an increasing weight given to the number of citations of each topic (i.e., models from PA to PC).

Table S8. Models and sub-models for computing topic's importance for a Phase 2 clinical trial.

Model	Overall	Stakeholder group specific (i)
**Mod-Pa**	$I = \frac{0.3m}{m_{max}} + \frac{0.35j}{j_{max}} + \frac{0.35u}{u_{max}} + \frac{0.1\gamma}{\gamma_{max}}$	$I = \frac{0.3m_i}{m_{i,max}} + \frac{0.35j_i}{j_{i,max}} + \frac{0.35u_i}{u_{i,max}}$
**Mod-Pb**	$I = \frac{0.4m}{m_{max}} + \frac{0.3j}{j_{max}} + \frac{0.3u}{u_{max}} + \frac{0.1\gamma}{\gamma_{max}}$	$I = \frac{0.4m_i}{m_{i,max}} + \frac{0.3j_i}{j_{i,max}} + \frac{0.3u_i}{u_{i,max}}$
**Mod-Pc**	$I = \frac{0.5m}{m_{max}} + \frac{0.25j}{j_{max}} + \frac{0.25u}{u_{max}} + \frac{0.1\gamma}{\gamma_{max}}$	$I = \frac{0.5m_i}{m_{i,max}} + \frac{0.25j_i}{j_{i,max}} + \frac{0.25u_i}{u_{i,max}}$

Supplementary results

Priority score

The priority score of topics divided by the type of stakeholders is presented in **Table S9** for policy makers and governmental institutions, **Table S10** for donors and funders, **Table S11** for NGOs, and **Table S12** for research institutions.

Table S9. Topics emerged with the discussion with policymakers and governmental institutions and corresponding level of emergency and importance, and the priority score computed using different models.

						Priority score		
**#[1]**	**Topic**	**Type**	**Urgency**	**Importance**	**No. citations**	**Mod-PA**	**Mod-PB**	**Mod-PC**
**1**	Disseminate CT information	Collaboration	Medium	High	13	0.88	0.90	0.92
**2**	CT sites	CT elements	High	Medium	12	0.86	0.87	0.88
**3**	Coordination among consortia	Collaboration	High	High	6	0.84	0.78	0.73
**4**	Access and pricing	Access & uptake	Medium	High	7	0.74	0.72	0.69
**5**	Country uptake	Access & uptake	Medium	High	7	0.74	0.72	0.69
**6**	Operational research	Access & uptake	Low	High	8	0.65	0.65	0.64
**7**	WHO requirements	Access & uptake	High	High	2	0.75	0.66	0.58
**8**	Regulatory requirements	Access & uptake	High	Medium	4	0.68	0.62	0.57
**9**	Special population	CT elements	High	High	1	0.72	0.63	0.54
**10**	TPP	CT elements	High	High	1	0.72	0.63	0.54
	CT design	CT elements	High	Medium	3	0.65	0.59	0.53
	Adherence	CT elements	High	Low	5	0.58	0.55	0.53
	Data quality	CT elements	High	Medium	1	0.61	0.53	0.46
	Economic analysis	CT elements	Medium	High	1	0.61	0.53	0.46
	AI/ML	CT elements	High	Low	3	0.54	0.49	0.45
	Biomarkers	CT elements	High	Low	2	0.51	0.46	0.41
	Coordination between CAGs	Collaboration	Medium	Medium	1	0.49	0.43	0.37
	Essential drug list	Access & uptake	Low	High	1	0.49	0.43	0.37
	FDC	Access & uptake	Low	High	1	0.49	0.43	0.37
	Capacity building	Access & uptake	Medium	Low	2	0.40	0.36	0.33
	Phase-out and phase-in	Access & uptake	Low	Medium	1	0.37	0.33	0.29
	DR emergence	Access & uptake	Low	Low	2	0.28	0.26	0.24
	Phase 3	Access & uptake	Low	Low	1	0.26	0.23	0.21

[1] Topics are ranked based on model Mod-PC

Table S10. Topics emerged with the discussion with donors and funders and corresponding level of emergency and importance, and the priority score computed using different models.

#[2]	Topic	Type	Urgency	Importance	No. citations	Priority score		
						Mod-PA	Mod-PB	Mod-PC
**1**	Access and pricing	Access & uptake	Medium	High	8	0.77	0.75	0.72
**2**	Country uptake	Access & uptake	Medium	High	6	0.72	0.68	0.65
**3**	Coordination among consortia	Collaboration	High	High	3	0.77	0.69	0.62
**4**	TPP	CT elements	High	High	2	0.75	0.66	0.58
**5**	Disseminate CT information	Collaboration	Medium	High	4	0.68	0.62	0.57
**6**	Special population	CT elements	High	High	1	0.72	0.63	0.54
**7**	WHO requirements	Access & uptake	High	High	1	0.72	0.63	0.54
**8**	CT sites	CT elements	High	Medium	1	0.61	0.53	0.46
**9**	Data quality	CT elements	High	Medium	1	0.61	0.53	0.46
**10**	Economic analysis	CT elements	Medium	High	1	0.61	0.53	0.46
	Regulatory requirements	Access & uptake	High	Medium	1	0.61	0.53	0.46
	Operational research	Access & uptake	Low	High	3	0.54	0.49	0.45
	Adherence	CT elements	High	Low	2	0.51	0.46	0.41
	FDC	Access & uptake	Low	High	2	0.51	0.46	0.41
	Essential drug list	Access & uptake	Low	High	1	0.49	0.43	0.37
	Capacity building	Access & uptake	Medium	Low	1	0.37	0.33	0.29
	Phase-out and phase-in	Access & uptake	Low	Medium	1	0.37	0.33	0.29
	DR emergence	Access & uptake	Low	Low	2	0.28	0.26	0.24

[2] Topics are ranked based on model Mod-PC

Table S11. Topics emerged with the discussion with non-governmental organizations and corresponding level of emergency and importance, and the priority score computed using different models.

						Priority score		
**#[3]**	**Topic**	**Type**	**Urgency**	**Importance**	**No. citations**	**Mod-PA**	**Mod-PB**	**Mod-PC**
**1**	Country uptake	Access & uptake	Medium	High	6	0.72	0.68	0.65
**2**	Disseminate CT information	Collaboration	Medium	High	6	0.72	0.68	0.65
**3**	Access and pricing	Access & uptake	Medium	High	5	0.70	0.65	0.61
**4**	Coordination among consortia	Collaboration	High	High	2	0.75	0.66	0.58
**5**	TPP	CT elements	High	High	2	0.75	0.66	0.58
**6**	WHO requirements	Access & uptake	High	High	2	0.75	0.66	0.58
**7**	CT sites	CT elements	High	Medium	4	0.68	0.62	0.57
**8**	Special population	CT elements	High	High	1	0.72	0.63	0.54
**9**	CT design	CT elements	High	Medium	2	0.63	0.56	0.49
**10**	Regulatory requirements	Access & uptake	High	Medium	2	0.63	0.56	0.49
	Data quality	CT elements	High	Medium	1	0.61	0.53	0.46
	Economic analysis	CT elements	Medium	High	1	0.61	0.53	0.46
	Microbiology biobank	CT elements	High	Medium	1	0.61	0.53	0.46
	Operational research	Access & uptake	Low	High	3	0.54	0.49	0.45
	Biomarkers	CT elements	High	Low	2	0.51	0.46	0.41
	Adherence	CT elements	High	Low	1	0.49	0.43	0.37
	Coordination with professional organizations	Collaboration	Medium	Medium	1	0.49	0.43	0.37
	FDC	Access & uptake	Low	High	1	0.49	0.43	0.37
	Phase 3	Access & uptake	Low	Low	1	0.26	0.23	0.21

[3] Topics are ranked based on model Mod-PC

Table S12. Topics emerged with the discussion with research institutions and corresponding level of emergency and importance, and the priority score computed using different models.

						Priority score		
**#[4]**	**Topic**	**Type**	**Urgency**	**Importance**	**No. citations**	**Mod-PA**	**Mod-PB**	**Mod-PC**
**1**	CT sites	CT elements	High	Medium	4	0.68	0.62	0.57
**2**	Coordination among consortia	Collaboration	High	High	1	0.72	0.63	0.54
**3**	Disseminate CT information	Collaboration	Medium	High	3	0.65	0.59	0.53
**4**	Access and pricing	Access & uptake	Medium	High	1	0.61	0.53	0.46
**5**	Country uptake	Access & uptake	Medium	High	1	0.61	0.53	0.46
**6**	CT design	CT elements	High	Medium	1	0.61	0.53	0.46
**7**	Regulatory requirements	Access & uptake	High	Medium	1	0.61	0.53	0.46
**8**	Biomarkers	CT elements	High	Low	1	0.49	0.43	0.37
**9**	Phase 3	Access & uptake	Low	Low	2	0.28	0.26	0.24

[4] Topics are ranked based on model Mod-PC
